# Validation of Cut-Points for Evaluating the Intensity of Physical Activity with Accelerometry-Based Mean Amplitude Deviation (MAD)

**DOI:** 10.1371/journal.pone.0134813

**Published:** 2015-08-20

**Authors:** Henri Vähä-Ypyä, Tommi Vasankari, Pauliina Husu, Ari Mänttäri, Timo Vuorimaa, Jaana Suni, Harri Sievänen

**Affiliations:** 1 The UKK Institute for Health Promotion Research, Tampere, Finland; 2 Haaga-Helia University of Applied Sciences, Vierumäki, Finland; University of St Andrews, UNITED KINGDOM

## Abstract

**Purpose:**

Our recent study of three accelerometer brands in various ambulatory activities showed that the mean amplitude deviation (MAD) of the resultant acceleration signal performed best in separating different intensity levels and provided excellent agreement between the three devices. The objective of this study was to derive a regression model that estimates oxygen consumption (VO2) from MAD values and validate the MAD-based cut-points for light, moderate and vigorous locomotion against VO_2_ within a wide range of speeds.

**Methods:**

29 participants performed a pace-conducted non-stop test on a 200 m long indoor track. The initial speed was 0.6 m/s and it was increased by 0.4 m/s every 2.5 minutes until volitional exhaustion. The participants could freely decide whether they preferred to walk or run. During the test they carried a hip-mounted tri-axial accelerometer and mobile metabolic analyzer. The MAD was calculated from the raw acceleration data and compared to directly measured incident VO_2_. Cut-point between light and moderate activity was set to 3.0 metabolic equivalent (MET, 1 MET = 3.5 ml · kg^-1^ · min^-1^) and between moderate and vigorous activity to 6.0 MET as per standard use.

**Results:**

The MAD and VO_2_ showed a very strong association. Within individuals, the range of r values was from 0.927 to 0.991 providing the mean r = 0.969. The optimal MAD cut-point for 3.0 MET was 91 mg (milligravity) and 414 mg for 6.0 MET.

**Conclusion:**

The present study showed that the MAD is a valid method in terms of the VO_2_ within a wide range of ambulatory activities from slow walking to fast running. Being a device-independent trait, the MAD facilitates directly comparable, accurate results on the intensity of physical activity with all accelerometers providing tri-axial raw data.

## Introduction

According to physical activity recommendations healthy adults need moderate intensity aerobic physical activity (PA) for a minimum of 150 or vigorous intensity PA for a minimum of 75 min on three days each week accumulating from PA bouts of at least 10 minutes [1]. Moderate intensity activities have energy expenditure between 3.0 to 5.9 metabolic equivalents (MET) and vigorous intensity activities higher than 6.0 METs. One MET is defined as the resting metabolic rate for quietly sitting and is about 3.5 ml · kg^-1^ · min^-1^, when expressed as oxygen consumption (VO_2_) rate [2]. Then 3.0 MET and 6.0 MET physical activities correspond 10.5 ml · kg^-1^ · min^-1^ and 21.0 ml · kg^-1^ · min^-1^ VO_2_, respectively.

Accelerometry provides a useful and feasible method to characterize PA during free living conditions. It permits objective measurements of the intensity, duration and frequency of daily PA and exercise [3] including assessment of short activity bouts that cannot be captured with questionnaires, interviews or diaries [4, 5]. However, the main challenges in accelerometry pertain to the use of proprietary algorithms [6, 7], and lack of valid physiologically determined cut-points for intensity of PA [8, 9], which both compromise the cross-study comparisons. Storing and processing the raw acceleration data have been proposed as the means to improve the comparability between different devices and studies [10].

We recently developed a novel method for universal analysis of PA from raw tri-axial accelerometer data [11]. In that study, raw acceleration data were collected during various sedentary and ambulatory activities and analysed with several classifiers in both time and frequency domain. Of these the mean amplitude deviation (MAD) of the resultant acceleration signal consistently provided the best performance in separating different PA intensity levels from each other. Most importantly the MAD enabled a direct comparison between the results of different accelerometer brands despite clearly different technical specifications of these devices.

The MAD value describes the mean value of the dynamic acceleration component. It is calculated from the resultant value of the measured tri-axial acceleration, which comprises both tri-axial dynamic components due to velocity changes and static component due to gravity. The static component is removed from the analysed time period (epoch) and the remaining dynamic component is rectified. The MAD value is the mean of the rectified signal within the epoch and independent of static component.

There is a strong correlation between incident MAD values and heart rate both among adults [11] and adolescents [12]. We therefore hypothesized that the incident MAD value also correlates strongly with actual VO_2_ at individual level. Previously similar acceleration signal-derived traits have been evaluated during treadmill walking and running and found to have strong correlation with VO_2_ [13–15] and be better predictor of VO_2_ than heart rate [16]. However treadmill derived relationship may overestimate the intensity of PA [17, 18], thus, accurate comparison is best achieved during actual locomotion.

The present study was carried out to develop a MAD-based model for predicting VO_2_ during locomotion within a wide range of speeds and to determine its accuracy. The main objective was to determine the MAD-based universal cut-points for light, moderate and vigorous PA corresponding to commonly accepted MET values for these intensity levels [1].

## Methods

### Participants

The study group consisted of 29 healthy volunteers, 15 males and 14 females. Prior to testing body height, weight and waist circumference were measured with standard methods (Table 1). Participants were informed of the experimental test protocol and they gave their written informed consent. This study conformed to the code of Ethics of the World Medical Association (Declaration of Helsinki) and it was approved by the Ethics Committee of Pirkanmaa Hospital District (R13040).

**Table 1:** Characteristics of the study group (mean ± SD).

Age (years)	35 ± 11
Height (cm)	172.1 ± 9.9
Weight (kg)	69.7 ± 12.4
Waist circumference (cm)	80 ± 9

### Test procedure

Participants performed a pace-conducted non-stop test on a 200 m long oval indoor track with slightly banked bends. Pace was verified by a so called “light rabbit” system, which comprises light sources alongside of the track. The light sources were installed at every 2 meters and they automatically lited-up one at a time according to the required pace. Every light source was also visible to tester. So participant’s location on the track could be determined with the accuracy of 2 meters. Initial speed was 0.6 m/s and it was increased by 0.4 m/s at every 2.5 minutes. The direction of travel was counter-clockwise. The participants had to keep up with the light sources and they could freely decide whether they preferred walking or running. The selected gait type was recorded for each stage. The test was continued until volitional exhaustion when the participant could not keep up with the lights anymore. End point was visually inspected by the tester and time at the end point was recorded.

### Measurements

A tri-axial accelerometer (Hookie AM20, Traxmeet Ltd, Espoo, Finland) was attached to the hip-mounted elastic belt at the level of the iliac crest. The accelerometer had ± 16 *g* measurement range and acceleration was measured at 100 Hz sampling frequency. Because the indoor track has banked turns only to left, it might have effect on the accelerometer output. Thus the accelerometer was placed either at the right (r-hip) or left side (l-hip) of the hip and the side was randomly selected. The left side (i.e. inner side of curve) was assigned to 13 participants and the right side (i.e. outer side of curve) to 16 participants. In addition, all participants carried one accelerometer in the middle of the back (mid) to emulate a situation where misplacement was the longest in terms of the preferred lateral location.

During the test procedure, VO_2_ was continuously measured with a portable breath-by-breath mobile metabolic analyser (Oxycon, Carefusion, Yorba Linda, CA, USA) and the data were recorded with a telemetry system. The metabolic cart was calibrated before each test according the manufacturer’s instructions.

### Data analysis

The analysis of the acceleration signal was based on the MAD value described recently [11].

Tri-axial acceleration was measured in raw mode from all three orthogonal measurement axes in actual g-units and stored for further analysis. In short, each measurement point (*i*) consisted of samples *x*_*i*_, *y*_*i*_ and *z*_*i*_. The resultant acceleration (*r*_*i*_), which defines the magnitude of the acceleration vector and contains both dynamic and static component of acceleration, was calculated for each (i) time point as

$${\text{r}}_{\text{i}}=\sqrt{{\text{x}}_{\text{i}}^{2}+{\text{y}}_{\text{i}}^{2}+{\text{z}}_{\text{i}}^{2}}$$ (1)

The epoch length used in the analysis was 6 s, and for each analysed epoch the mean resultant value (*R*_*ave*_) describing the static component of acceleration was calculated as

$$R_{ave}=\;\frac{1}{\text{N}}\sum_{\text{i}=\text{j}}^{\text{j+N-1}}{\text{r}}_{\text{i}}$$ (2)

The MAD value of the given epoch was calculated as

$$\text{MAD}=\frac{1}{\text{N}}\sum_{\text{i}=\text{j}}^{\text{j+N-1}}\left|{\text{r}}_{\text{i}}-{\text{R}}_{\text{ave}}\right|$$ (3)

where *N* is the number of samples in the epoch (ie, 600) and *j* is the start point of the epoch. The unit of the MAD is milligravity (m*g*); ie, the Earth’s gravity 1*g* is equal to 1000 m*g*.

To allow the participants to find the steady rhythm the acceleration was analysed for the final 2 minutes of each stage. As a measure of steady-stage VO_2_ for given speed mean VO_2_ of the final minute of the corresponding stage was used. VO_2peak_ value was the highest measured VO_2_ for one minute period during the test. Measured end time made it possible to calculate the mean speed during the final two and half minutes of the test (v_max_). The v_max_ value depends on the maximum speed achieved during the test and time it has been maintained. The oxygen cost of movement (ml · kg^-1^ · km^-1^) was calculated for each stage as the ratio of measured VO_2_ (ml · kg^-1^ · min^-1^) to known speed (km/min).

### Statistical methods

Data were analysed with SPSS 21.0 (SPSS Science, Chicago, USA) software. First, independent two-sample t-test was performed to investigate whether the MAD values were different between right and left side for each speed. In addition, Pearson correlation between VO_2_ and the MAD was determined for each participant. Mean correlation coefficient was calculated by first z-transforming the individual correlation coefficients, taking the arithmetic mean of transformed coefficients, and then by back-transforming the mean. The generalized linear model was used to estimate VO_2_. Since the data were not normally distributed, gamma regression model was used. Incident VO_2_ was the dependent variable and the incident MAD value, physical characteristics (age, weight, height, and waist circumference) and performance values (VO_2peak_ and v_max_) served as the independent predictor variables. Furthermore, because the data during walking were normally distributed, the estimation of the VO_2_ during walking from the MAD value was based on linear regression model. Stages with respiratory exchange ratio over 1.0 or not fully completed were excluded from the analysis.

To find optimal intensity based cut-points for the MAD values the receiver operator characteristics (ROC) analysis was used. The measured VO_2_ values were used as a golden standard. VO_2_ values were converted to MET values by using the standard conversion factor (1 MET = 3.5 ml · kg^-1^ · min^-1^) and performances were classified to light (< 3.0 MET), moderate (3.0–5.9 MET) and vigorous (> 6.0 MET) activity. For both 3 MET and 6 MET limits a new dichotomous variable was created to define the outcome of the test. If a measured MET value was less than limit, the outcome was negative and the variable value was set to 0. Otherwise the outcome was positive and the variable was set to 1. After that ROC curve analysis were conducted to determine sensitivity and specificity values. Sensitivity and specificity defines correctly identified positive and negative values. The MAD value which maximized the sum of the specificity and sensitivity was selected as the optimal cut-point. Also the area under curve (AUC) value was determined. AUC value of 1 indicates a perfect classifier whereas AUC value of 0.5 denotes no discriminatory value [19].

## Results

**Fig 1:**
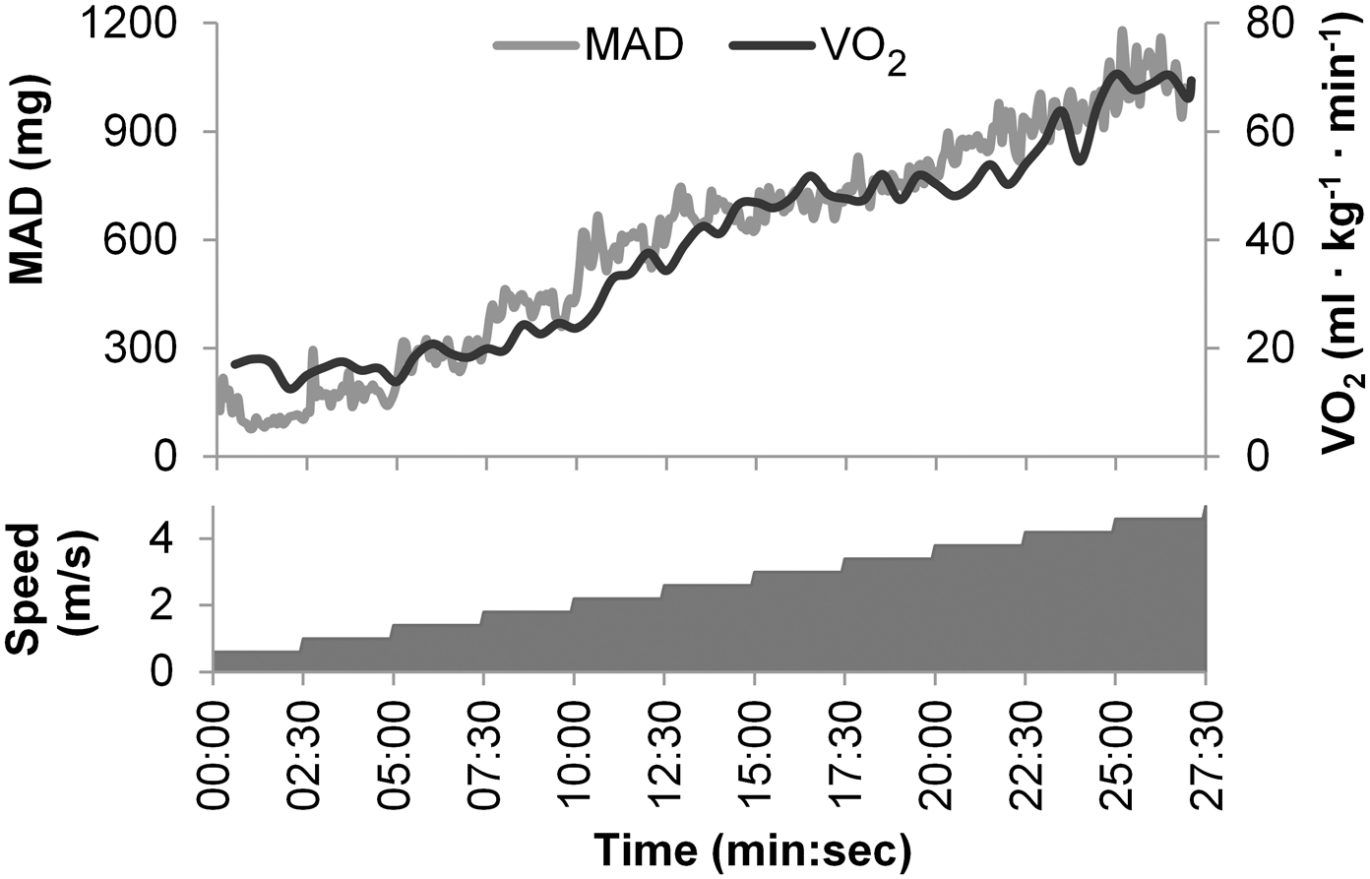
Illustration of pace conducted test performance. The lower graph describes the required speed and the upper curves the measured VO_2_ and MAD values during the whole test. The unit mg denotes milligravity.

The Fig 1 illustrates one test performance, where the participant walked the first four stages and changed to running at stage five (2.2 m/s). In the beginning of the test there was some trouble in achieving steady pace, which can be seen from MAD and VO_2_ curves. The required pace was maintained almost 27 minutes.

**Fig 2:**
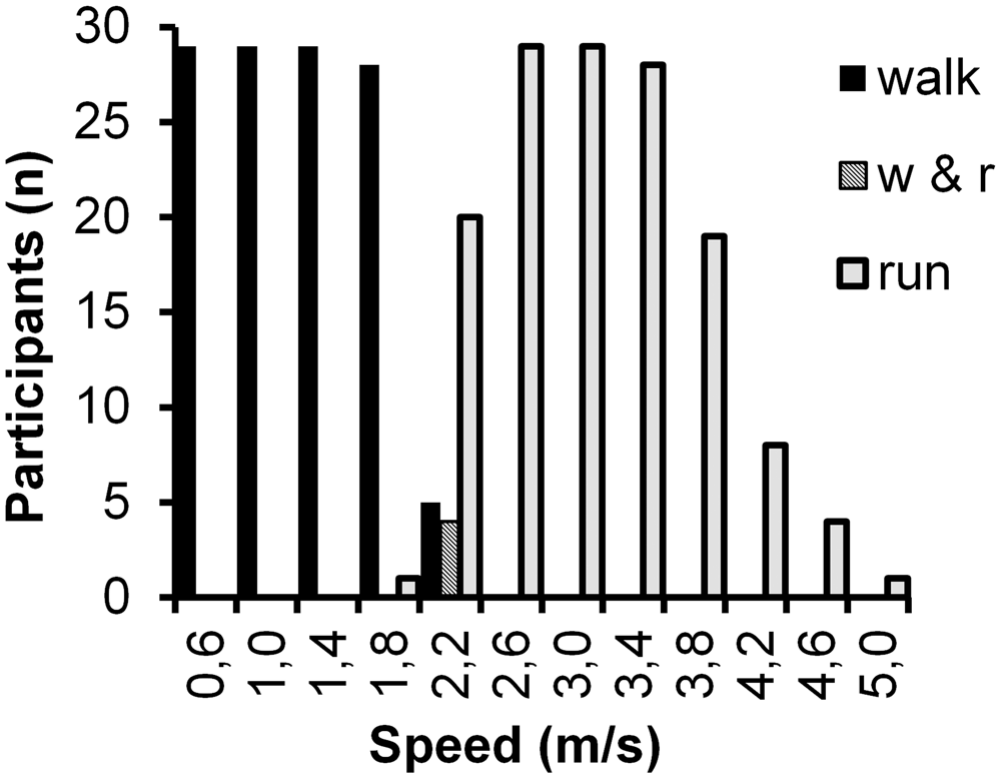
Preferred gait types in different speeds. The preferred gait of the fully completed stages is shown for each speed.

**Fig 3:**
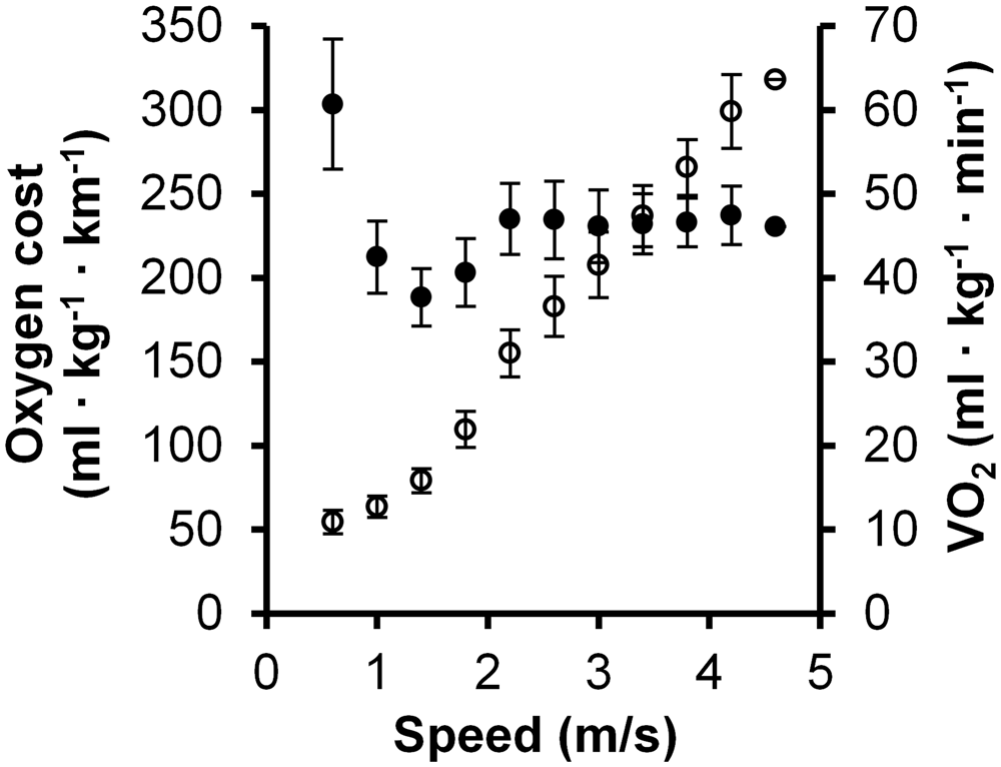
Oxygen cost and consumption in different speeds. The oxygen cost of the locomotion (black circles) describes the economy and the oxygen consumption (open circles) the intensity of the movement.

The Fig 2 shows the number of fully completed stages and the preferred gait. Typically the participants changed the gait type (from walking to running) at the beginning of the stage. At speed 2.2 m/s (7.9 km/h) four participants changed the gait type in the middle of the stage. The mean VO_2peak_ was 56.0 ± 7.1 ml · kg^-1^ · min^-1^ (range 45–69 ml · kg^-1^ · min^-1^). The range of the v_max_ was 3.1–5.1 m/s (11.1 km/h– 18.4 km/h). At the group level the oxygen cost of locomotion reached the minimum at the speed 1.4 m/s (Fig 2). During running, after initial increase the energy cost remained quite steady despite increasing speed. At the individual level the curve of the oxygen cost was u-shaped both for walking and running. For walking, 26 participants have the lowest oxygen cost at speed 1.4 m/ (5.0 km/h), while for running the minimum value varied between speed from 2.2 m/s (7.9 km/h) to 3.8 m/s (13.7 km/h). VO_2_ showed a curvilinear increase during walking and linear during running with increasing speed (Fig 3). The highest MAD and VO_2_ value for the stage containing barely walking was 651 m*g* and 30.2 ml · kg^-1^ · min^-1^, whereas the lowest value for stage containing barely running was 581 m*g* and 26.1 ml · kg^-1^ · min^-1^, respectively.

### Sensor placement and MAD

**Fig 4:**
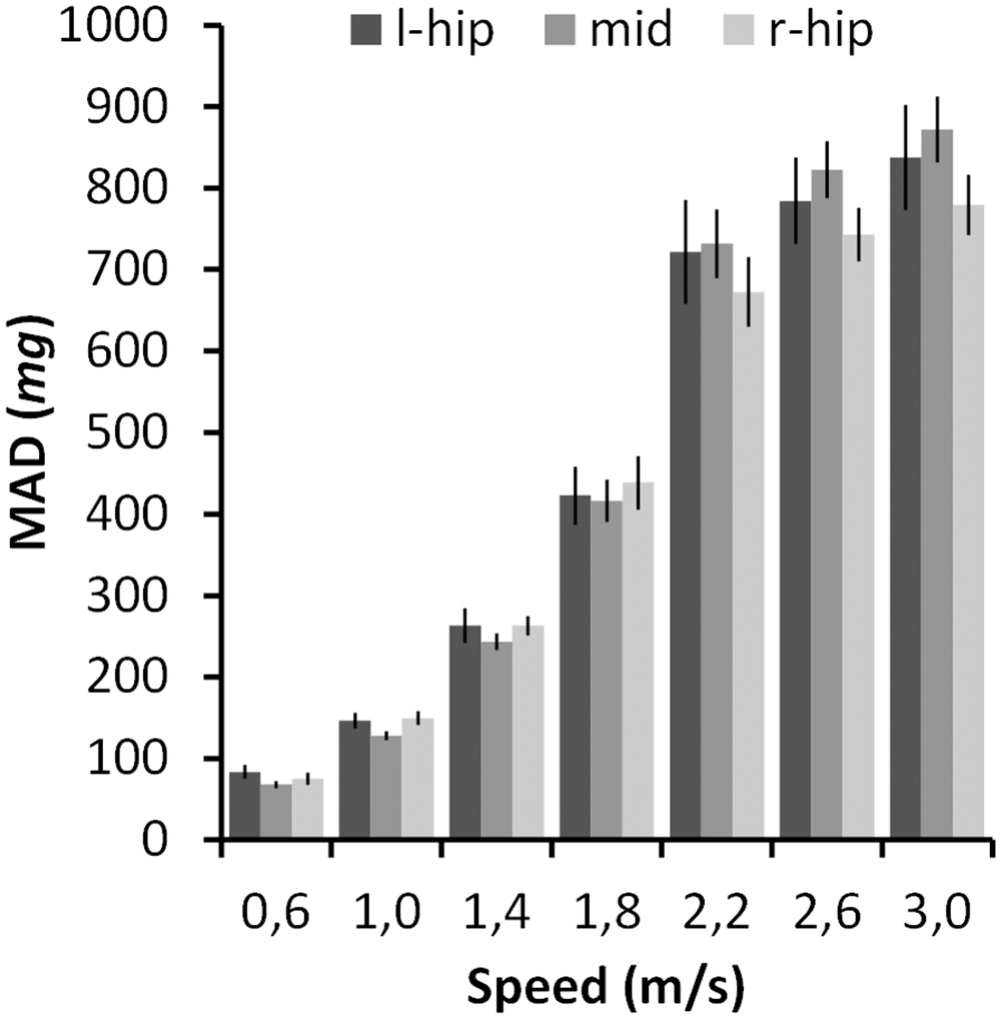
Sensor placement and MAD in different speeds. The measured mean MAD value with the three sensor positions is shown only for speeds performed by all 29 participants. The whiskers denote the 95% confidence intervals of the mean value. The unit m*g* denotes milligravity.

Sensor placement conferred a slight effect on measured MAD values, the effect being largest during running (Fig 4). With slow speed walking the mid position values and with running the right-side position values were slightly lower. The MAD values increased with increasing gait speed.

### Individual correlations

**Fig 5:**
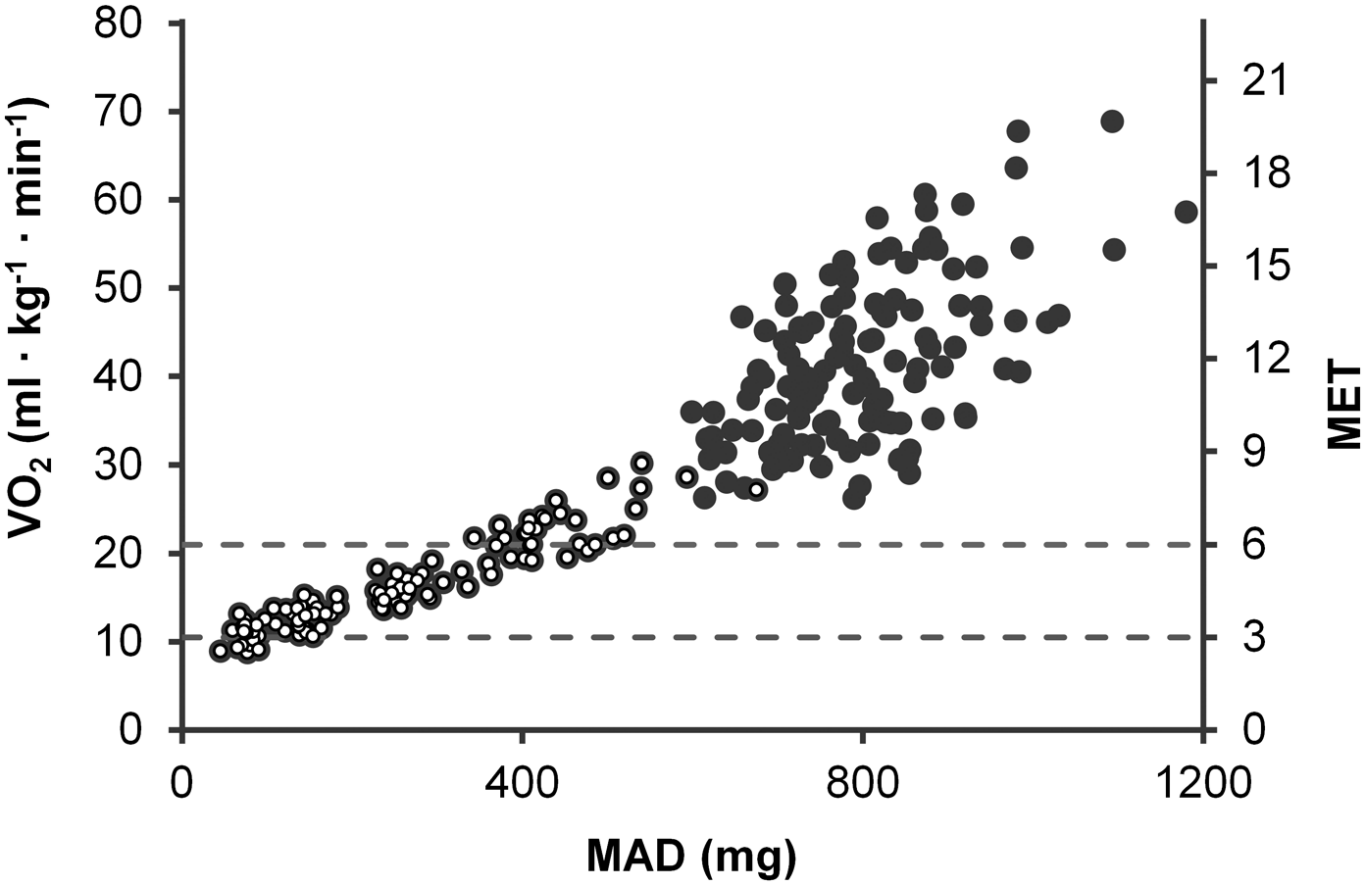
Relationship between VO_2_ and MAD. Stages containing only walking have open circle and other stages black circle. The dotted lines denote 3 MET and 6 MET thresholds and the unit m*g* denotes milligravity.

Within individuals, the correlations between both MAD and VO_2_, and MAD and speed were very high (Table 2). In all participants, the MAD value increased with increasing VO_2_ or speed for both walking and running. Mean correlation values were highest for walking, and somewhat lower for data containing both walking and running, or running alone. This is due to different regression slopes between MAD and VO_2_, and MAD and speed during walking and running, and larger variation in data during running (Fig 5).

**Table 2:** Mean and range of within-individual correlations between MAD and VO_2_, and MAD and speed.

	MAD and VO_2_	MAD and speed
Both walk and run	0.975 (0.927–0.991)	0.969 (0.933–0.986)
Walking only	0.995 (0.976–1.000)	0.990 (0.962–1.000)
Running only	0.976 (0.850–0.999)	0.988 (0.886–0.999)

### Prediction models between MAD and VO_2_

The direct relationship between the incident MAD and VO_2_ values were estimated with following equation: VO_2_ (ml/kg/min) = 10.015 · e^0.0017 · MAD (mg)^ (r = 0.958, standard error of the estimate (SEE) = 6.05 ml/kg/min), where *mg* denotes milligravity (Fig 5). For walking only, the prediction model based on linear regression was: VO_2_ = 7.920 + 0.0331 · MAD (*mg*) (r = 0.943, SEE = 1.66 ml/kg/min) (Fig 5). By using all measured values the estimation equation was following: VO_2_ (ml/kg/min) = 2.351 · e^(0.00177 · MAD (mg)- 0.282 · vmax (m/s) + 0.0183 · VO2peak (ml · kg-1 · min-1) + 0.0117 · height (cm)– 0.0142 · weight (kg) + 0.00693 · waist circumference (cm)– 0.00211 · age (years))^ (r = 0.975, SEE = 4.46 ml/kg/min). Parameters in the equation are in the order of significance and for each parameter p-value was less than 0.05.

### Optimal cut-points

**Fig 6:**
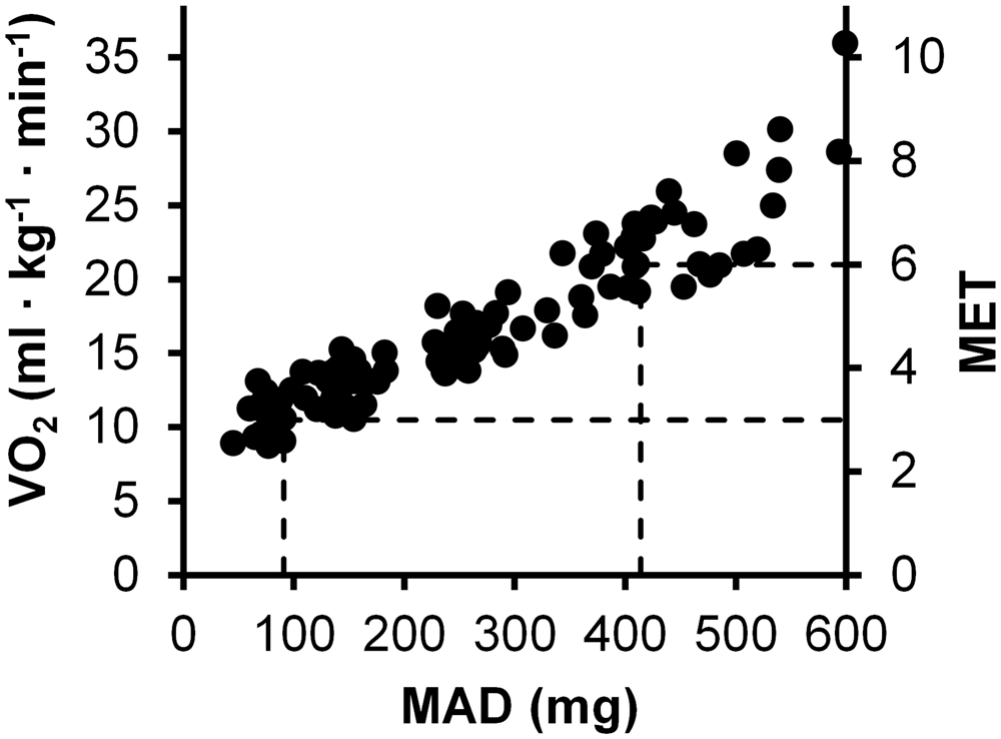
Optimal cut-points. The dotted lines represent the optimal MAD cut-points for 3 and 6 MET limits. The MAD values are shown up to 600 m*g*. The unit m*g* denotes milligravity.

**Fig 7:**
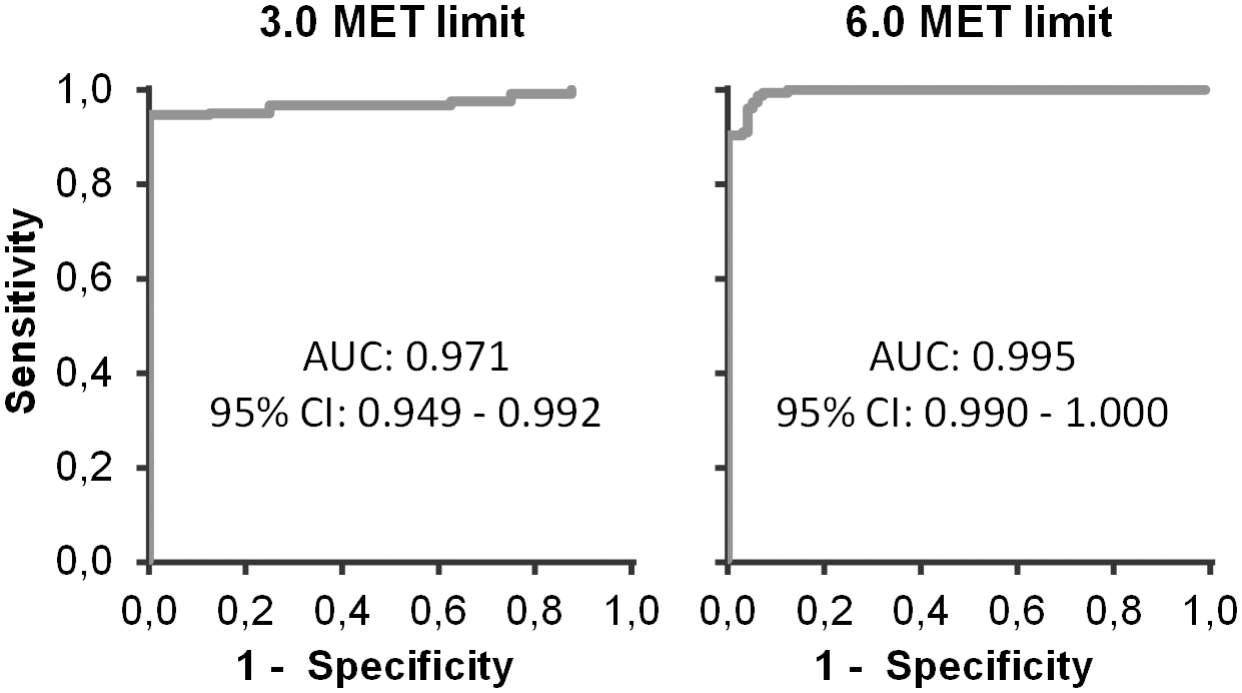
ROC curves and AUC for cut-points. ROC curve and AUC (mean and 95% confidence interval (95% CI)) for 3.0 MET limit (left) and for 6.0 MET limit (right).

According to the ROC curve analysis the optimal MAD cut-point for intensity of 3.0 MET was 91 *mg* and for 6.0 MET 414 *mg* (Fig 6). Sensitivity and specificity values were 100% and 96% for the 3.0 MET cut-point, and 96% and 95% for 6.0 the MET cut-point. The AUC and 95% confidence interval for both limits is shown in the Fig 7.

## Discussion

The present study demonstrated that the MAD is a highly valid method to estimate the intensity of PA within a wide range of locomotion from slow walking to fast running. The study also produced valid cut-points for accurate determination of light, moderate and vigorous intensity levels of PA that are much needed in epidemiological population studies. Because the calculation of the MAD is based on the raw acceleration data and has been shown to be device-independent, the MAD approach offers a possibility to obtain directly comparable and accurate results on the intensity of PA with all accelerometers which provide tri-axial raw data within a sufficient dynamic range [11].

The accuracy of the MAD method to predict VO_2_ during walking and running compares well with other commonly used methods [20–22]. As is the case with other methods, the accuracy was better for walking than running. However the MAD value is not compromised by the common ceiling effect, where the accelerometer output values reach a peak at a certain speed and do not increase in response to further speed increments [22–24]. In the present study increasing speed produced increasing MAD values in a dose-response manner for each participant.

The oxygen cost of the locomotion was at typical level for all except the first stage of the test (i.e. slow walking), when the cost was higher than expected. Apparently the emotional excitement at the beginning of the test contributed to relatively high measured VO_2_ values. Also some participants had difficulties in achieving a steady pace at the beginning of the first stage. The measured oxygen cost was 303 ± 39 ml/kg/km at the 0.6 m/s speed, while in the previous study the oxygen cost was below 250 ml/kg/km at the 0.67 m/s speed [25]. The most economical movement speed in the present study was 1.4 m/s with the oxygen cost of 188 ± 17 ml/kg/km which is line with other studies [25, 26]. Preferred gait transition speed from walking to running is slightly lower than energetically optimal transition speed [27, 28] and it seems to depend on various metabolic and biomechanical factors related to transition. In the present study preferred speed for gait transition was in line with literature [27, 28]. With running the oxygen cost was slightly lower for the higher speeds. This is probably consequence of the fact that only participants with good running economy reached the higher speeds and could maintain the speed for several minutes.

The association between the MAD and VO_2_ was very strong both at individual level and at group level. By adding individual anthropometric and performance data into the prediction model only a slight improvement in the estimation of VO_2_ could be attained, especially during running. However, when the MAD value was excluded, the most significant individual predictors were VO_2_peak and v_max_ values, which might be difficult to obtain in practise. The contribution of these predictors is apparently related to running economy and the bouncing characteristics of running. The mechanics and energetics of running depend on the kinetic and potential energy of the whole body and the body segments, besides storage and release of mechanical energy by the contracting muscles and tendons. The accelerometer cannot separate whether the energy for the speed change is produced actively by the muscle or passively by the tendon. The simpler mechanics and energetics characteristics of the walking seem to account for more straightforward estimation of VO_2_ with accelerometer [29, 30]. Nevertheless, the prediction of the incident VO_2_ was excellent whether or not the additional predictors were known, and importantly, the estimation was sufficient enough for accurate classification of PA in terms of light, moderate and vigorous intensity.

Previously it has been shown that sensor placement on either hip or waist area can have effect on accelerometer output [17]. With the MAD values the effect of sensor placement was marginal in relation to the wide range of MAD values in different speeds. However, some observations are worth discussing. First, at low walking speeds the mid position showed slightly lower MAD values than the other positions. This is explained by the sensor movement which is apparently higher on the side due to pelvis tilting. Second, during running the right side MAD values were slightly lower. This is attributable to the characteristics of the indoor track used in the present study. It had banked curves and participants moved only to anti-clockwise direction. The inner (left) and outer legs (right) apparently experienced somewhat asymmetrical loading in the curves. These observations not only show how sensitive method the accelerometry can be in detecting subtle differences in movements, but also underline the importance of keeping placement of the sensor as constant as possible.

Some analysis methods of accelerometry data can produce inaccurate results, if the sensor orientation in relation to gravity is not controlled for [31]. With the MAD method this is not a concern, as illustrated by the following examples. Assuming that the orientation of the sensor x-axis is perpendicular to ground (ie, parallel to the gravity vector) while both y- and z-axes are parallel to ground, then the measurement vector M = (x, y, z) is M = (1.000, 0.000, 0.000) in *g*-units. The resultant acceleration R is in this static situation equal to Earth’s gravity 1.000 *g*. If the sensor moved downwards with 0.5 g acceleration and thereafter upwards with 0.5 *g* acceleration then the sensor readings would be M = (0.500, 0.000, 0.000) and M = (1.500, 0.000, 0.000) and corresponding R values 0.500 *g* and 1.500 *g*. Both values deviate 0.500 *g* i.e. 500 *mg* from the static value. Assuming next that the sensor is rotated 45° around the z-axis resulting in M = (0.707, 0.707, 0) and R = 1.000 g in the static situation. For the above described dynamic 0.5 g downwards and upwards accelerations the sensor readings would be M = (0.354, 0.354, 0.000) and M = (1.061, 1.061, 0). Again, the corresponding R values are 0.500 *g* and 1.500 *g* and deviation from the static value is 0.500 *g* despite the different position of the sensor.

Another problem with accelerometers is their offset, which means the difference between the measured value of the accelerometer and the true acceleration. Although the sensors are calibrated during manufacturing process, the offset cannot be avoided, because it is sensitive to external conditions, like temperature [32]. Using the conditions of the previous example and assuming that all axes have 0.05 g offset the calculations are as follows: When the x-axis is perpendicular to ground, sensor readings for static condition would be M = (1.050, 0.050, 0.050) and R value 1.052 *g*. For the 0.5 *g* dynamic upward and downward accelerations the corresponding values are M = (0.550, 0.050, 0.050) and M = (1.550, 0.050, 0.050), and the R values 0.555 *g* and 1.552 *g* resulting in 0.499 *g* mean deviation from the static value. In the 45° rotated case the sensor readings for the static condition would be M = (0.757, 0.757, 0.050) and for dynamic conditions M = (0.404, 0.404, 0.050) and M = (1.111, 1.111, 0.050). The respective R values would be 1.072 *g*, 0.573 *g* and 1.572 *g* resulting in 0.499 *g* mean deviation from the static value. Inferred from the above described examples, the offset can have minor effect on the results but there is no need to use excessive methods to calibrate the sensor when the MAD approach is used.

Participant’s aerobic fitness in the present study was high as can be judged from their high VO_2max_ values. All but three participants reached the highest class in seven level fitness classifications [33] and the remaining three belonged to classes 5–6. This can be considered a strength of the study because a wide speed range of locomotion and oxygen consumption was attained from very slow walking to high speed running. Other strengths were the relatively large sample size and the direct measurement of the expiratory gases during actual bipedal movement on the track instead of a treadmill. The main weakness of the study was that no other activities than walking and running on the track were studied. On the other hand, locomotion is the most common type of physical activity among ordinary people.

## Conclusion

In conclusion, the MAD is a valid method to estimate the intensity of PA within a wide range of bipedal human locomotion. The MAD values highly reflect the incident oxygen consumption within a wide range of walking and running speeds. The proposed cut-points offer a valid base for assessing the health effects of PA. Sensor positioning does not compromise the results. This study further underscores the utility of the simple and universal MAD approach as the means to overcome the challenges for comparisons between studies and different accelerometers.

## Supporting Information

S1 File Measurement data. File contains measured oxygen consumption and MAD values for each stage.
(TXT)

## References

[1] United States Department of Health and Human Services. Physical Activity Guidelines for Americans Be Active, Healthy, and Happy! Washington: United States Department of Health and Human Services; 2008.

[2] Jetté M, Sidney K, Blümchen G. Metabolic equivalents (METS) in exercise testing, exercise prescription, and evaluation of functional capacity. Clin Cardiol. 1990 8;13(8):555–65. 2204507 10.1002/clc.4960130809

[3] Troiano RP, Berrigan D, Dodd KW, Mâsse LC, Tilert T, McDowell M. Physical activity in the United States measured by accelerometer Med Sci Sports Exerc. 2008 1;40(1):181–8. 18091006 10.1249/mss.0b013e31815a51b3

[4] Bonomi AG, Goris AH, Yin B, Westerterp KR. Detection of type, duration, and intensity of physical activity using an accelerometer. Med Sci Sports Exerc. 2009 9;41(9):1770–7 10.1249/MSS.0b013e3181a24536 19657292

[5] Esliger DW, Tremblay MS. Physical activity and inactivity profiling: the next generation. Can J Public Health. 2007;98(Suppl 2):195–207. 18213949

[6] Marschollek M. A semi-quantitative method to denote generic physical activity phenotypes from long-term accelerometer data—the ATLAS index. PLoS One. 2013 8;8(5):e63522 10.1371/journal.pone.0063522 23667631 PMC3648464

[7] Welk GJ, McClain J, Ainsworth BE. Protocols for evaluating equivalency of accelerometry-based activity monitors. Med Sci Sports Exerc. 2012 44(Suppl 1):S39–49. 22157773 10.1249/MSS.0b013e3182399d8f

[8] Freedson P, Bowles HR, Troiano R, Haskell W. Assessment of physical activity using wearable monitors: recommendations for monitor calibration and use in the field. Med Sci Sports Exerc. 2012 1;44(1 Suppl 1):S1–4. 10.1249/MSS.0b013e3182399b7e 22157769 PMC3245520

[9] Orme M, Wijndaele K, Sharp SJ, Westgate K, Ekelund U, Brage S. Combined influence of epoch length, cut-point and bout duration on accelerometry-derived physical activity. Int J Behav Nutr Phys Act. 2014 3 10;11(1):34 10.1186/1479-5868-11-34 24612726 PMC4008000

[10] Pasqui G, Bonomi AG, Westerterp KR. Daily physical activity assessed with accelerometers: new insights and validation studies. Obes Rev. 2013 14(6):451–62. 10.1111/obr.12021 23398786

[11] Vähä-Ypyä H, Vasankari T, Husu P, Suni J, Sievänen H. A universal, accurate intensity-based classification of different physical activities using raw data of accelerometer. Clin Physiol Funct Imaging. Epub 2014 Jan 7. 10.1111/cpf.12127 24393233

[12] Aittasalo M, Vähä-Ypyä H, Vasankari T, Husu P, Jussila A-M, Sievänen H. Mean amplitude deviation calculated from raw acceleration data: A novel method for classifying the intensity of adolescents' physical activity irrespective of accelerometer brand. In press 10.1186/s13102-015-0010-0 PMC4527117 26251724

[13] Bouten CV, Westerterp KR, Verduin M, Janssen JD. Assessment of energy expenditure for physical activity using a triaxial accelerometer. Med Sci Sports Exerc. 1994 12;26(12):1516–23. 7869887

[14] McGregor SJ, Busa MA, Yaggie JA, Bollt EM. High resolution MEMS accelerometers to estimate VO2 and compare running mechanics between highly trained inter-collegiate and untrained runners. PLoS One. 2009 10 6;4(10):e7355 10.1371/journal.pone.0007355 19806216 PMC2752199

[15] Gleiss A. C., Wilson R. P., & Shepard E. L. Making overall dynamic body acceleration work: on the theory of acceleration as a proxy for energy expenditure. Methods in Ecology and Evolution. 2011 2(1), 23–33.

[16] Halsey LG, Shepard EL, Hulston CJ, Venables MC, White CR, Jeukendrup AE, et al Acceleration versus heart rate for estimating energy expenditure and speed during locomotion in animals: tests with an easy model species, Homo sapiens. Zoology (Jena). 2008;111(3):231–41. 18375107 10.1016/j.zool.2007.07.011

[17] Yngve A, Nilsson A, Sjostrom M, Ekelund U. Effect of monitor placement and of activity setting on the MTI accelerometer output. Med Sci Sports Exerc. 2003 2;35(2):320–6 12569223 10.1249/01.MSS.0000048829.75758.A0

[18] Vanhelst J, Zunquin G, Theunynck D, Mikulovic J, Bui-Xuan G, Beghin L. Equivalence of accelerometer data for walking and running: treadmill versus on land. J Sports Sci. 2009 5;27(7):669–75. 10.1080/02640410802680580 19424900

[19] Fan J, Upadhye S, Worster A. Understanding receiver operating characteristic (ROC) curves. CJEM. 2006 1;8(1):19–20. 17175625 10.1017/s1481803500013336

[20] Freedson PS, Melanson E, Sirard J. Calibration of the Computer Science and Applications, Inc. accelerometer. Med Sci Sports Exerc. 1998 30(5):777–81. 9588623 10.1097/00005768-199805000-00021

[21] Fudge BW, Wilson J, Easton C, Irwin L, Clark J, Haddow O, et al Estimation of oxygen uptake during fast running using accelerometry and heart rate. Med Sci Sports Exerc. 2007 1;39(1):192–8. 17218902 10.1249/01.mss.0000235884.71487.21

[22] Crouter SE, Bassett DR Jr. A new 2-regression model for the Actical accelerometer. Br J Sports Med. 2008 (3):217–24. 17761786 10.1136/bjsm.2006.033399

[23] John D, Tyo B, Bassett DR. Comparison of four ActiGraph accelerometers during walking and running. Med Sci Sports Exerc. 2010 42(2):368–74. 10.1249/MSS.0b013e3181b3af49 19927022 PMC2809132

[24] Rowlands AV, Stone MR, Eston RG. Influence of speed and step frequency during walking and running on motion sensor output. Med Sci Sports Exerc. 2007 39(4):716–27. 17414811 10.1249/mss.0b013e318031126c

[25] Martin PE, Rothstein DE, Larish DD. Effects of age and physical activity status on the speed-aerobic demand relationship of walking. J Appl Physiol (1985). 1992 73(1):200–6. 1506370 10.1152/jappl.1992.73.1.200

[26] Cunningham DA, Rechnitzer PA, Pearce ME, Donner AP. Determinants of self-selected walking pace across ages 19 to 66. J Gerontol. 1982 9;37(5):560–4. 7096927 10.1093/geronj/37.5.560

[27] Hreljac A. Preferred and energetically optimal gait transition speeds in human locomotion. Med Sci Sports Exerc. 1993 25(10):1158–62. 8231761

[28] Sentija D, Markovic G. The relationship between gait transition speed and the aerobic thresholds for walking and running. Int J Sports Med. 2009 30(11):795–801. 10.1055/s-0029-1237711 19838979

[29] Willems PA, Cavagna GA, Heglund NC. External, internal and total work in human locomotion. J Exp Biol. 1995 198(Pt 2):379–93. 7699313 10.1242/jeb.198.2.379

[30] Cavagna GA. The landing-take-off asymmetry in human running. J Exp Biol. 2006 209(Pt 20):4051–60. 17023599 10.1242/jeb.02344

[31] Qasem L, Cardew A, Wilson A, Griffiths I, Halsey LG, Shepard EL, et al Tri-axial dynamic acceleration as a proxy for animal energy expenditure; should we be summing values or calculating the vector? PLoS One. 2012;7(2):e31187 10.1371/journal.pone.0031187 22363576 PMC3281952

[32] van Hees VT, Fang Z, Langford J, Assah F, Mohammad A, da Silva IC, et al Autocalibration of accelerometer data for free-living physical activity assessment using local gravity and temperature: an evaluation on four continents. J Appl Physiol (1985). 2014 10 1;117(7):738–44. 25103964 10.1152/japplphysiol.00421.2014 PMC4187052

[33] Shvartz E, Reibold RC. Aerobic fitness norms for males and females aged 6 to 75 years: a review. Aviat Space Environ Med. 1990 61(1):3–11. 2405832

